# The influence of thirty-degree leg elevation on noradrenaline requirements administered as a prophylactic variable infusion during cesarean delivery, an open-label randomized controlled trial

**DOI:** 10.1186/s44158-025-00290-7

**Published:** 2025-10-28

**Authors:** Mina Adolf Helmy, Nader N. Naguib, Kerlous Adolf Helmy, Lydia Magdy Milad

**Affiliations:** 1 Department of Anesthesia and Critical Care Medicine, Faculty of Medicine, Cairo University, Cairo, Egypt; 2 Department of Obstetrics, Gynecology and Perinatology, Sechenov University, Moscow, Russia

**Keywords:** Leg elevation, Spinal anesthesia-induced hypotension, Noradrenaline, Cesarean section, Maternal hemodynamics

## Abstract

**Background:**

Spinal anesthesia is the preferred technique for elective cesarean delivery; however, it is frequently associated with spinal anesthesia-induced hypotension. To mitigate this, prophylactic vasopressors have become a cornerstone of obstetric anesthesia practice. Despite their use, hypotension may still occur, prompting the exploration of adjunctive maneuvers to enhance hemodynamic stability and reduce vasopressor requirements. This study hypothesized that passive leg elevation would reduce the need for noradrenaline during cesarean delivery under spinal anesthesia.

**Methods:**

In this randomized controlled trial, we evaluated the effect of 30-degree leg elevation on noradrenaline requirements. Noradrenaline was administered as a variable infusion, ranging from 0.05 to 0.14 µg/kg/min. Participants were randomly assigned to either the control group or the leg elevation group. The primary outcome was the average noradrenaline requirement in each group.

**Results:**

A total of 80 healthy pregnant patients were included in the final analysis, with 40 patients in each group. The mean noradrenaline requirement was significantly lower in the leg elevation group compared to the control group (0.067 ± 0.01 vs. 0.079 ± 0.01 µg/kg/min, respectively; *p* < 0.05). Additionally, the incidence of hypotension was reduced in the leg elevation group (20%) compared to the control group (40%).

**Conclusion:**

Among healthy parturients undergoing elective cesarean section, passive leg elevation significantly reduced noradrenaline requirements and was associated with a lower incidence of hypotension. This simple maneuver may serve as a valuable adjunct to pharmacologic prophylaxis in spinal anesthesia.

**Trial registration:**

The study was registered by the principal investigator (M. Helmy) at ClinicalTrials.gov under the identifier NCT06822699 on February 7, 2025.

## Highlights

• This is the first randomized trial to assess the impact of leg elevation on noradrenaline requirements.

• Leg elevation resulted in a lower incidence of spinal anesthesia-induced hypotension.

• Noradrenaline requirements were lower in the leg elevation group compared to the control group.

## Introduction

Spinal anesthesia-induced hypotension (SAIH), defined as a decrease in systolic blood pressure (SBP) greater than 20% from baseline, is a frequent and critical complication during cesarean delivery, with reported incidence rates reaching up to 80% in some studies [1]. If not promptly recognized and managed, SAIH can result in severe maternal and neonatal consequences, including compromised uteroplacental perfusion and neonatal acidosis. Therefore, early diagnosis and aggressive management are essential to mitigate these risks [1, 2].

The use of prophylactic vasopressors to prevent SAIH is a well-established and widely accepted practice in obstetric anesthesia. As part of current best practice, vasopressors are routinely recommended to support maternal hemodynamics and minimize adverse outcomes [1]. In this context, noradrenaline (norepinephrine) has emerged as a favorable option due to its minimal side effect profile and superior hemodynamic characteristics [2]. Compared to phenylephrine, noradrenaline better preserves heart rate and cardiac output. Furthermore, a recent study concluded that noradrenaline has demonstrated approximately 13-fold greater potency than phenylephrine [3]. However, higher infusion doses may pose risks, including cardiac dysrhythmias and rebound hypertension [2, 4], necessitating careful titration.

In addition to pharmacologic strategies, mechanical interventions have shown promise. A previous study [5] reported that 30-degree leg elevation reduced the incidence of SAIH, although prophylactic vasopressors were not employed in that context. Similarly, leg compression has been associated with reduced phenylephrine infusion requirements, suggesting that lower limb interventions may enhance hemodynamic stability [6]. Based on these findings, we hypothesized that leg elevation would reduce noradrenaline infusion requirements in pregnant patients undergoing elective cesarean delivery. To the best of our knowledge, this is the first study to explore the effect of leg elevation on prophylactic noradrenaline administration.

## Patients and methods

This randomized controlled trial was conducted at a university hospital following approval from the institutional Research Ethics Committee of the Faculty of Medicine, Cairo University (Kasr Al Ainy Hospital) under approval number N-90–2025, and was registered at ClinicalTrials.gov (NCT06822699). The study was conducted over 7 weeks, from March 14, 2025, to May 8, 2025. Randomization was performed using computer-generated numbers and concealed using opaque closed envelopes. The envelopes were opened by a research assistant who was not involved in this study. This study was reported in accordance with the CONSORT standards. Each patient signed an informed consent form before the commencement of the study, and we randomly allocated 83 singleton pregnant patients (20–40 years). Inclusion criteria comprised healthy parturients classified as ASA II with singleton pregnancies scheduled for elective cesarean delivery under spinal anesthesia. Exclusion criteria included hypertensive disorders of pregnancy (chronic hypertension, gestational hypertension, and preeclampsia), patient refusal, baseline SBP < 100 mmHg, non-standard indications for cesarean delivery (e.g., maternal request without medical indication), and contraindications to spinal anesthesia. Additional exclusions were applied to cases with inadequate spinal block height, defined as sensory levels below T4. Demographic data, including age, gestational age, weight, and body mass index (BMI), were recorded. Upon arrival in the operating room (OR), an 18-gauge cannula was inserted into a forearm vein. Standard monitors (electrocardiography, pulse oximetry, and noninvasive blood pressure) were applied. The blood pressure cuff was applied to the opposite arm of the intravenous (IV) access, and blood pressure was measured at 1-min intervals until delivery and every 5 min thereafter. Baseline SBP was determined by calculating the average of three consecutive blood pressure measurements with <10% variation before anesthesia, with the patient lying supine with a 15-degree left lateral tilt, and 60%, 80%, 90%, and 120% were calculated according to baseline values.

### Anesthesia protocol

Spinal anesthesia was administered with the patient in the sitting position at the L4–L5 interspace using a 25-gauge Quincke-type spinal needle (Kindly Medical Devices Co., Ltd., China). The L4–L5 interspace was identified through surface anatomical landmarks, notably by palpating the iliac crests to delineate Tuffier’s line, which generally corresponds to the level of the L4 vertebral body or the L4–L5 interspace. A standardized intrathecal regimen consisting of 10 mg of hyperbaric bupivacaine (Sunny Pivacaine; Bupivacaine HCl 20 mg/4 mL, Sunny Medical, Cairo, Egypt) and 25 µg of fentanyl (Fentanyl-Hameln; 100 µg/2 mL, Hameln Pharmaceuticals, Germany) was delivered to achieve a sensory block at the T4 dermatome. The target sensory level was assessed 5 min after intrathecal administration and before surgical incision, using bilateral evaluation of cold sensation loss along the thoracic dermatomes. This was performed by gently applying an alcohol-soaked cotton swab to the skin.

### Fluid and noradrenaline administration protocol

While administering spinal anesthesia, all participants received a co-load of 200 mL of lactated Ringer’s solution. In addition, warmed lactated Ringer’s solution was continuously infused at a rate of 10 mL·kg^1^·h^−1^ throughout the surgical procedure. No preloading was administered to any participant. A noradrenaline administration (Norepinephrine-Mirola, 4 mg/4 mL International Advanced Pharmaceutical Industries “INAD pharma” for Mirola, Cairo, Egypt) was diluted in 500 mL glucose 5% to give a final concentration of 8 μg/mL and administered according to the following protocol: an initial bolus of 0.15 µg/kg was administered, followed by a continuous infusion at 0.05 µg/kg/min. The infusion rate was subsequently increased in increments of 0.03 µg/kg/min if SBP decreased by 10%, 20%, and ≥30%, reaching 0.08, 0.11, and 0.14 µg/kg/min, respectively. In cases of severe hypotension, defined as SBP < 60% of baseline, an additional bolus of 5 µg was given. When SBP returned to within 10% of baseline, the infusion rate was readjusted to 0.05 µg/kg/min. If hypertension occurred, defined as SBP > 120% of baseline, the noradrenaline infusion was temporarily discontinued until SBP returned to within 10% of the baseline value. Bradycardia was defined as a heart rate of less than 60 beats per minute and was treated with atropine (0.5 mg). This stepwise escalation schedule was based on pilot observations and supported by previous studies [2, 4, 7]. The maximum infusion rate of 0.14 µg/kg/min was selected based on previous literature, which confirmed its safety profile in obstetric and critical care settings [7].

Notably, SBP was reassessed every 1 min following each noradrenaline dose adjustment to prevent over-titration. Furthermore, all anesthesiologists received standardized training, which included hands-on simulation and competency checklists, to ensure consistent application of the titration algorithm and minimize inter-operator variability.

### Patient allocation

**Fig. 1:**
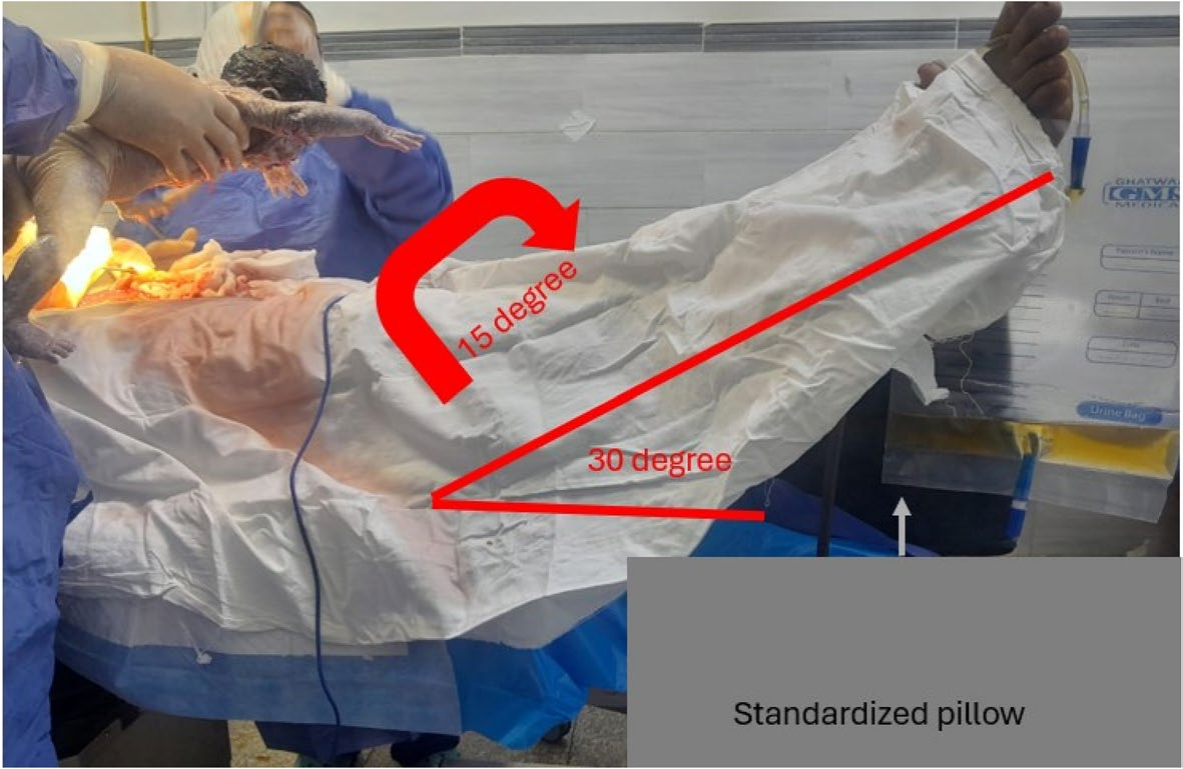
Illustrative image depicting the patient positioned supine with legs elevated approximately 30 degrees using a standardized pillow. A 15-degree left uterine tilt is achieved by angling the operating table to the left

Following spinal anesthesia, participants were randomly assigned to two groups: a leg elevation group (*n* = 40) and a control group (*n* = 40). In the leg elevation group, a standardized pillow with a height of 30 cm was placed beneath the heels to achieve an elevation angle of approximately 30°. To achieve a 15-degree left uterine displacement, the operating table was adjusted accordingly (Fig. 1). This positioning was initiated immediately after spinal anesthesia and maintained throughout the procedure. The combined positioning technique was selected to enhance venous return and support hemodynamic stability while minimizing interference with surgical access and preserving operator ergonomics. Table height was adjusted as needed to prevent disruption of the procedural workflow.

Patients in the control group were positioned supine with a 15-degree left uterine displacement.

Notably, all patients received the same 15-degree left uterine tilt, ensuring that aortocaval compression did not influence the incidence of hypotension observed between groups. This tilt was achieved solely by adjusting the inclination of the operating table, without physically repositioning the patient, and was consistently applied across both groups. To avoid residual aortocaval compression and ensure consistent positioning, a 15-degree left lateral tilt was confirmed using a digital inclinometer (Baseline® 12–1075, USA), zeroed before each measurement, and placed on the operating table surface prior to patient positioning.

### Data collection and study outcomes

Data collection included blood pressure, heart rate, APGAR score, and umbilical artery pH at 5 min. The primary outcome variable of this study is the average noradrenaline requirement (mcg/kg/min), defined as the total noradrenaline administered as an infusion plus boluses divided by patient weight and infusion time. Other outcomes included the overall incidence of hypotension (defined as SBP < 80% of baseline), severe hypertension (defined as SBP < 60% of baseline), time to first hypotensive episode, bradycardia, reactive hypertension (defined as SBP > 120% of baseline), APGAR score, and umbilical artery pH at 5 min.

### Sample size

The sample size was calculated using G*Power, version 3.1.9.4 (Heinrich Heine University Düsseldorf, Düsseldorf, Germany). We conducted a pilot study that included five patients and revealed a mean ± standard deviation (SD) noradrenaline requirement of 0.08 ± 0.01 μg/kg/min. We assumed that a 10% reduction in average noradrenaline requirements would be clinically significant for study power and an alpha error of 95% and 0.05, respectively. The minimum total sample size was 68 patients, and the sample size was increased to 80 patients to compensate for possible dropouts.

Notably, the SD of noradrenaline requirement observed in the final study (0.01 μg/kg/min in both groups) was consistent with the SD reported in the pilot study, thereby validating the assumptions used in our sample size calculation.

### Statistical analysis

Normality of the data was checked using the Shapiro–Wilk test. Data are reported as median and quartile or mean and SD values, as appropriate. Unpaired sample *t*-tests were used to compare normally distributed variables, whereas skewed data were analyzed using the Mann–Whitney test. For comparisons of categorical data, the chi-square test was used. However, when expected cell frequencies were <5 in 2 × 2 contingency tables, Fisher’s exact test was applied to ensure statistical validity. A 2-way ANOVA used to analyze serial SBP changes incorporated a mixed-effects model framework, with patient identifier included as a random effect to account for repeated within-subject measurements over time appropriately. Statistical analysis was conducted using IBM SPSS Statistics, version 27 (IBM Corp., Armonk, NY, USA). Statistical significance was set at *p* < 0.05.

## Results

**Fig. 2:**
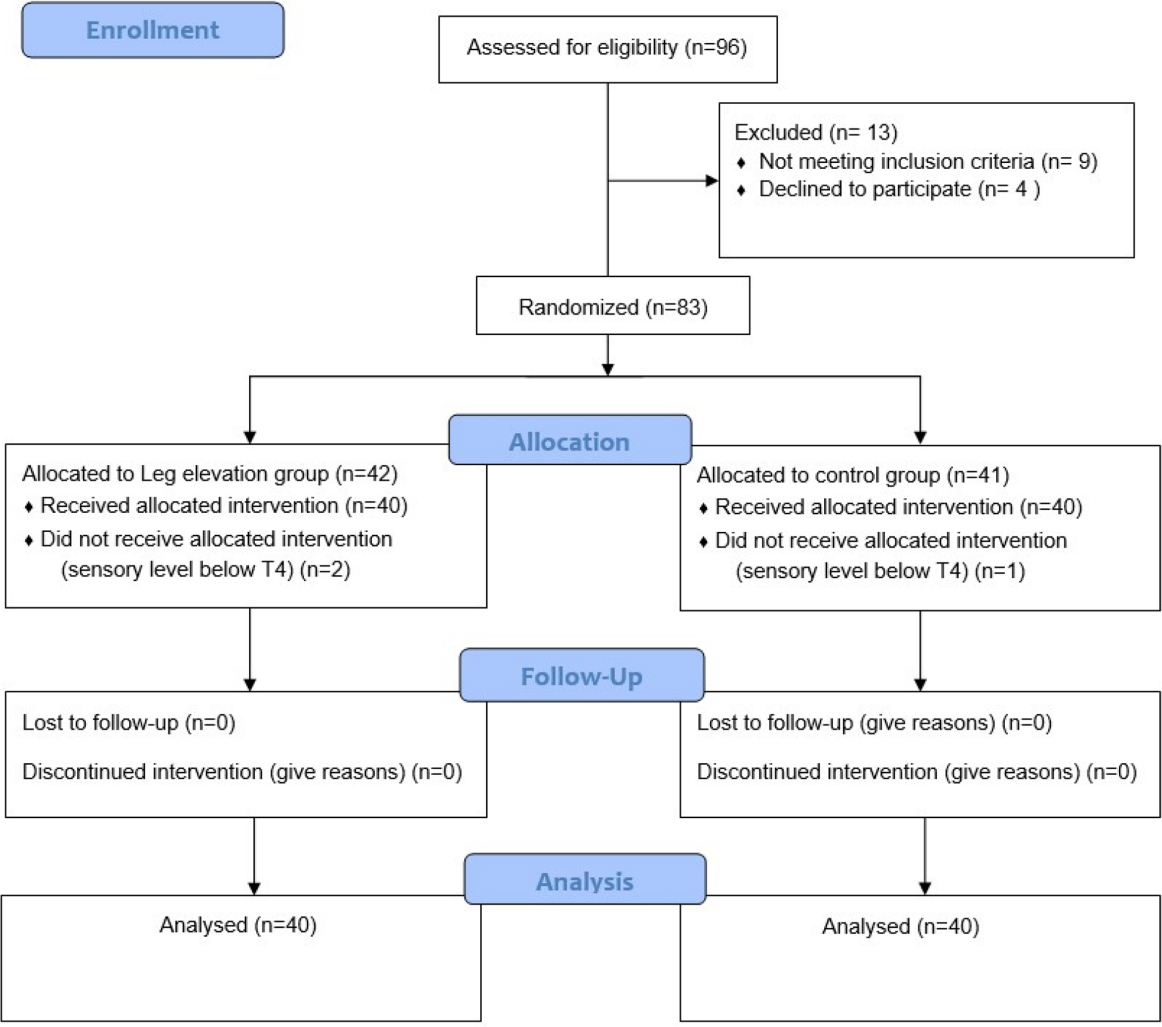
Patient enrollment

A total of 96 patients were screened for eligibility; 16 were excluded based on predefined criteria. Eighty patients were ultimately included in the final analysis, with 40 patients allocated to each group (Fig. 2). Baseline demographic and clinical characteristics, including age, weight, gestational age, baseline heart rate, SBP, estimated blood loss, and time to delivery, were comparable between the leg elevation (LE) and control groups (Table 1).

**Table 1:** Demographic data

	Leg elevation (*n* = 40)	Control group (*n* = 40)	*P* value
Age (years)	28.5 ± 5.1	30.1 ± 5	0.174
Weight (kg)	71.5 ± 9.2	71.4 ± 9.8	0.963
BMI (kg/m^2^)	28.4 ± 2.2	28.2 ± 3.2	0.755
Gestational age (weeks)	38.2 ± 0.8	38.0 ± 0.9	0.263
Baseline HR (beats/min)	83 ± 11	84 ± 10	0.584
Baseline SBP (mmHg)	125 ± 5	123 ± 6	0.167
Induction to delivery time (minutes)	16.4 ± 2	17.6 ± 2.2	0.672
Duration of procedure (minutes)	41.6 ± 4	40.2 ± 4.2	0.151
EBL (mL)	730 ± 136	731 ± 143	0.955

Data presented as mean ± standard deviation

*BMI* body mass index, *EBL* estimated blood loss, *HR* heart rate, *SBP* systolic blood pressure

**Fig. 3:**
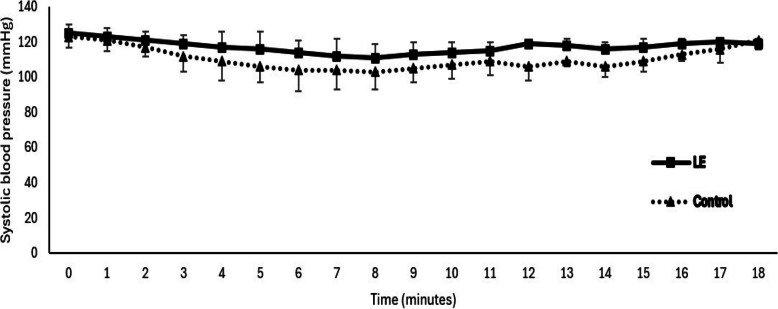
Systolic blood pressure over time. LE: leg elevation

The total noradrenaline requirement was significantly lower in the LE group compared to the control group (0.067 ± 0.01 vs. 0.079 ± 0.01 µg/kg/min, respectively; *p* < 0.05) (Table 2). The LE group also demonstrated a reduced incidence of overall hypotension (20%) and severe hypotension (2.5%) compared to the control group (Table 2). SBP decreased in both groups following spinal anesthesia, with a more pronounced decline observed in the control group (Fig. 3).

**Table 2:** Maternal and neonatal outcomes

	Leg elevation (*n* = 40)	Control group (*n* = 40)	*P* value
Time to first episode (minutes)	6 (2.3–6.8)	4.5 (3–7.5)	0.951
Incidence of overall hypotension	8/40 (20%)	16/40 (40%)	0.043^*^
Incidence of severe hypotension	1/40 (2.5%)	8/40 (20%)	0.029^*^
Hypotensive episodes			
One	6/40 (15%)	9/40 (22.5%)	
Two	2/40 (5%)	6/40 (15%)	0.192
Three	0/40 (0%)	1/40 (2.5%)	
Hypertension	1/40 (2.5%)	0/40 (0%)	>0.999
Bradycardia	0/40 (0%)	1/40 (2.5%)	>0.999
Total noradrenaline requirements (μg)	78 ± 16	95 ± 24	<0.001^*^
Total noradrenaline requirements (μg/kg/min)	0.067 ± 0.01	0.079 ± 0.01	<0.001^*^
5-min APGAR score	9 (9–10)	9 (8.3–10)	0.790
Umbilical artery pH at 5 min	7.29 (7.24–7.31)	7.26 (7.21–7.29)	0.013^*^
Umbilical artery PCO2 (mmHg)	48.5 (22–55)	51.5 (48–57)	0.087
Umbilical artery BE (mmol/L)	−1 (−2 to +1)	−3 (−4 to +1)	0.078
NICU admission (*n*, %)	0/40 (0%)	0/40 (0%)	>0.999

Data presented as mean ± standard deviation, median (quartiles), and count (percentage)

*BE* base excess, *NICU* neonatal intensive care unit

^*^Denotes statistical significance

Neonatal outcomes were comparable between groups. The 5-min APGAR scores, umbilical artery PCO_2_, and base excess values did not differ significantly. Although the umbilical artery pH was statistically higher in the leg elevation group compared to the control group (7.29 vs. 7.26, *p* = 0.013), both values remained within the normal physiological range. Importantly, this difference did not reflect clinical significance, as no neonates in either group required admission to the neonatal intensive care unit. These findings further support the safety of the intervention (Table 2).

Leg elevation at 30 degrees was successfully maintained throughout all procedures. The mean duration of surgery was 41.6 ± 4.0 min. No maternal discomfort or pressure-related complaints were reported, and no requests for repositioning were made by the surgical team. Operating table height readjustment was required in 2 cases (5%) due to minor ergonomic preferences expressed by the operating surgeon. Specifically, the 30-degree leg elevation slightly altered the lower limb position relative to the surgical field, prompting a downward adjustment of table height (approximately 5–7 cm) to optimize hand positioning and visibility. This adjustment was performed pre-incision and did not interfere with the anesthetic protocol or necessitate repositioning of the patient. These findings suggest that the maneuver was well tolerated and did not interfere with the surgical workflow (Table 3).

**Table 3:** Feasibility outcomes related to 30-degree leg elevation

Duration of surgery (minutes)	41.6 ± 4
Leg elevation maintenance throughout (*n*, %)	40/40 (100%)
Maternal discomfort (*n*, %)	0/40 (0%)
Pressure-related complaints (*n*, %)	0/40 (0%)
Need for table height readjustment (*n*, %)	2/40 (5%)
The surgical team requested repositioning (*n*, %)	0/40 (0%)

Data presented as mean ± standard deviation and count (percentage)

## Discussion

The current study demonstrated that passive leg elevation significantly reduces noradrenaline requirements during cesarean delivery under spinal anesthesia. Furthermore, the incidence of both overall hypotension and severe hypotension was lower in the leg elevation group compared to the control group.

Spinal anesthesia induces sympathectomy in the anesthetized regions, leading to vasodilation and subsequent hypotension, a physiological response that can have serious consequences if not promptly recognized and managed [8, 9]. The use of prophylactic vasopressors to prevent spinal anesthesia-induced hypotension (SAIH) is a well-established and widely endorsed practice in obstetric anesthesia [1]. As part of current best practice guidelines, vasopressors are routinely recommended to maintain maternal hemodynamic stability and reduce the risk of adverse maternal and neonatal outcomes. Noradrenaline has emerged as a valuable agent in this context, offering effective prevention of SAIH [2, 4]. Moreover, prior dose-finding studies have identified norepinephrine dosing in obstetric anesthesia as mild (0.01–0.05 µg/kg/min or 4–6 µg bolus), moderate (0.06–0.1 µg/kg/min or 6–10 µg bolus), and high (>0.1 µg/kg/min or >10 µg bolus) [10–12]. This classification provides a framework for evaluating vasopressor use and supports the clinical relevance of interventions. However, vasopressor administration is not without risk, as it may lead to rebound hypertension and cardiac dysrhythmias.

The noradrenaline escalation protocol we adopted, starting at 0.05 µg/kg/min and increasing by 0.03 µg/kg/min up to 0.14 µg/kg/min, was based on pilot observations and supported by literature. Sheng et al. [7] demonstrated that variable-rate infusion up to 0.14 µg/kg/min improves hemodynamic stability without increasing the risk of arrhythmia or hypertension, reinforcing both the safety and effectiveness of the dosing strategy during cesarean delivery.

Several adjunctive strategies have been proposed to reduce vasopressor requirements, including pneumatic leg compression and compression stockings [6, 13]. Passive leg raising is another such intervention, promoting autotransfusion of blood from the lower extremities to the central circulation [5], thereby enhancing blood pressure. A radioisotope scan study demonstrated that a 20-degree leg elevation resulted in a 34% redistribution of blood volume toward the central compartment [14]. This maneuver also increases left ventricular preload and cardiac output, contributing to improved hemodynamic stability [15]. The selection of 30-degree leg elevation was based on pilot observations indicating that this angle provided a greater hemodynamic response than lower elevations while remaining feasible within the surgical environment. Mechanistically, leg elevation enhances venous return and increases preload, which may be particularly effective when combined with noradrenaline. Unlike pure α-agonists such as phenylephrine, noradrenaline possesses β-adrenergic activity that helps preserve cardiac output. This pharmacodynamic profile, when paired with improved central blood volume from leg elevation, may synergistically reduce the incidence of post-spinal hypotension more effectively than either intervention alone.

This is the first study to specifically evaluate the impact of leg elevation on noradrenaline requirements during spinal anesthesia for elective cesarean delivery. Our findings align with those of a previous study that reported a reduced incidence of SAIH with leg elevation [5]. However, that study did not utilize prophylactic vasopressors, administered a preload of 20 mL/kg lactated Ringer’s solution, and maintained leg elevation only until skin incision, highlighting key methodological differences. The results of the current study indicate that leg elevation may serve as a safe and effective non-pharmacologic intervention to enhance hemodynamic stability during cesarean delivery. Although the absolute reduction in noradrenaline requirements between groups was modest, the findings suggest that leg elevation is a safe, simple, and non-pharmacologic maneuver that can reduce vasopressor use without compromising maternal safety. Additionally, leg elevation was associated with a lower overall incidence of hypotension (20% vs. 40%) and a substantial reduction in severe hypotension (2.5% vs. 20%), further supporting its potential utility in routine clinical practice, particularly in resource-limited settings or among patients with increased sensitivity to vasopressors. Our study has some limitations. First, it was conducted at a single center with a relatively small sample size, which may limit the generalizability of the findings. Second, the open-label design introduces a theoretical risk of selection bias; however, the demographic characteristics of the enrolled cohort were comparable across groups. Third, we included only healthy pregnant patients, and thus, future research is needed to evaluate the effect of leg raising in patients with comorbidities. Fourth, although a standardized 30-cm pillow was used to approximate a 30-degree leg elevation, individual angles were not measured. Due to variations in leg length and BMI, the actual elevation angle likely differed between patients, introducing some variability in intervention intensity. Therefore, future research should consider individualized angle adjustment or direct measurement to improve consistency and reduce outcome variance. Finally, although the 30-degree leg elevation was maintained throughout the entire cesarean section in our study, we acknowledge that this positioning may pose practical challenges in broader clinical settings, particularly among patients with higher BMI or restricted mobility. While no ergonomic concerns or intraoperative adjustments apart from table height adjustment were reported by our surgical team, these findings may not be fully generalizable to populations with different anthropometric profiles. Therefore, future studies are warranted to assess the feasibility and clinical impact of sustained leg elevation in more diverse patient populations, especially those with elevated BMI and limited mobility.

## Conclusion

Among healthy singleton pregnant patients undergoing elective cesarean delivery, a 30-degree leg elevation was associated with a reduction in noradrenaline requirements. Furthermore, the incidence of both overall hypotension and severe hypotension was lower in the leg elevation group compared to the controls.

## Data Availability

The data that support the findings of this study are available from the corresponding author upon reasonable request.
